# 27-Hydroxycholesterol Promotes the Transfer of Astrocyte-Derived Cholesterol to Neurons in Co-cultured SH-SY5Y Cells and C6 Cells

**DOI:** 10.3389/fcell.2020.580599

**Published:** 2020-11-24

**Authors:** Yushan Wang, Xiaona Zhang, Tao Wang, Wen Liu, Lijing Wang, Ling Hao, Mengwei Ju, Rong Xiao

**Affiliations:** School of Public Health, Beijing Key Laboratory of Environmental Toxicology, Capital Medical University, Beijing, China

**Keywords:** 27-hydroxycholesterol, cholesterol transport, cholesterol elimination, endoplasmic reticulum, plasma membrane, CYP46A1 translocation

## Abstract

Abnormality in cholesterol homeostasis in the brain is a feature of Alzheimer’s disease (AD). 27-Hydroxycholesterol (27-OHC) has been identified as a possible biomarker of AD, but its effects on cholesterol metabolism have not been fully characterized. This study was aimed to investigate the impacts of 27-OHC on cholesterol metabolism in nerve cells. SH-SY5Y cells and C6 cells were co-cultured and treated with 5, 10, and 20 μM 27-OHC for 24 h. Results showed that 27-OHC decreased cholesterol levels and up-regulated the expression of transport-related proteins in C6 cells. In SH-SY5Y cells, 27-OHC increased cholesterol accumulation, especially on plasma membrane (PM), which was consistent with the up-regulation of expressions of cholesterol endocytosis receptors, lipid raft-related proteins, and cholesterol esterase. Simultaneously, accumulation of membrane cholesterol promoted cholesterol conversion to 24S-OHC by CYP46A1(24S-hydroxylase) transfer from the endoplasmic reticulum (ER) to PM. Besides, Aβ levels were elevated in SH-SY5Y cells after 27-OHC treatment. Our results suggest that 27-OHC motivates the transfer of astrocyte-derived cholesterol to neurons. Although there exists a feedback mechanism that excessive cholesterol promotes its conversion to 24S-OHC, the increased cholesterol induced by 27-OHC could not be wholly offset in neurons.

## Introduction

Dysregulation in brain cholesterol homeostasis has lately been considered to be closely related to the development of neurodegenerative diseases such as Alzheimer’s disease (AD), Parkinson’s disease (PD), Huntington’s disease (HD), Niemann’s disease, etc. (Martin et al., 2010; Maulik et al., 2013; Zhang and Liu, 2015). Elevated cholesterol levels in the central nervous system (CNS), especially the accumulation in neurons sensitizes the brain to AD-like pathologies (Djelti et al., 2015). It is widely known that cholesterol is essential for maintaining membrane fluidity, signal transduction, and participating in lipid raft formation to regulate the co-localization of neurotransmitters and receptors involved in neuronal synaptic function (Lee et al., 2014), while AD-related pathologies including tau and Aβ abnormalities have both direct or indirect connection with cholesterol (van der Kant et al., 2019). Changes in cholesterol metabolism have been demonstrated to promote tau hyperphosphorylation and subsequent aggregations (Gamba et al., 2012). In addition, cholesterol accumulation in membrane raft domains aids in Aβ production from APP through co-localization of the involved proteins resulting in multiple cellular and molecular dysfunctions, neurological decline, and cell death, gradually progressing to the irreversible atrophy of brain tissue (Kojro et al., 2001; Grimm et al., 2005; Sole-Domenech et al., 2013).

Cholesterol synthesis and transport in the brain are regulated by endogenous mechanisms, as the blood–brain barrier (BBB) is impermeable to cholesterol-containing lipoprotein particles, preventing cholesterol in the peripheral circulation from entering the brain (Moutinho et al., 2016). *In vitro* studies demonstrated that astrocytes are mainly responsible for synthesizing cholesterol in the brain and releasing cholesterol-rich ApoE lipoprotein complex by ATP binding cassette transporter proteins (mainly ABCA1 and ABCG1) (Pfrieger and Ungerer, 2011; Chen et al., 2013). The lipoproteins are taken up by neurons via dedicated receptors such as LDLR, LRP1, and SR-B1, then release free cholesterol (FC) through a series of hydrolysis in endosomes and lysosomes (Pfrieger and Ungerer, 2011). The spare cholesterol could be stored as cholesterol ester (CE) formed by acyl-coenzyme-A cholesterol acyltransferase (ACAT) (Brown and Jessup, 2009). Besides, another mechanism for excessive cholesterol clearance from neurons is its conversion into 24(S)-hydroxycholesterol (24S-OHC), a vital cholesterol hydroxylated metabolite capable of readily crossing the BBB and entering the circulation to be further metabolized in the liver (Moutinho et al., 2016; Kacher et al., 2019). This conversion is catalyzed by cholesterol 24-hydroxylase encoded by the CYP46A1 gene uniquely expressed in neurons (Moutinho et al., 2016; Petrov and Pikuleva, 2019).

Recently, many lines of epidemiological investigations manifesting dysregulated levels of blood lipid profile, which may be caused by unbalanced diets, support the notion that abnormally high blood cholesterol levels might contribute to disease progression during aging (Ma et al., 2017; Ahmed et al., 2018; Power et al., 2018). Moreover, the shreds of evidence from animal models have confirmed the impairment of brain structure and function caused by altered circulating cholesterol levels (Ledreux et al., 2016; Chen et al., 2017). Considering the relative independence pathway of cholesterol metabolism in the peripheral and CNS caused by BBB, certain cholesterol oxidation products, such as 27-hydroxycholesterol (27-OHC), which is capable of moving in and out of the brain, become the research focus of connecting the two cholesterol metabolic pools (Leoni and Caccia, 2011; Zmyslowski and Szterk, 2019). 27-OHC is an enzymatic production of cholesterol catalyzed by mitochondrial cytochrome P450 sterol 27-hydroxylase (CYP27A1) enzyme, which is mainly expressed in the liver. Our previous published studies had reported that the increased level of 27-OHC in the brain induced by dietary cholesterol played a key role in devastating the learning and memory function in rats (Zhang et al., 2015, 2018). *In vitro* studies, 27-OHC exhibited toxic effects in neurons by contributing to lysosomal membrane permeabilization-mediated pyroptosis (Chen et al., 2019). Furthermore, 27-OHC exerted a potential impact on regulating cholesterol synthesis and transport in astrocytes (An et al., 2017). Collectively, our results provided new insights by which AD-related pathological changes induced by cholesterol metabolism are regulated by 27-OHC, thus paving ways for revealing the pathological mechanism of neurodegenerative diseases. However, it remains to be fully understood how 27-OHC affects the circulation of cholesterol between astrocytes and neurons as well as the metabolism of cholesterol in neurons. Herein we used co-cultured C6 cells and SH-SY5Y cells to explore the effects of 27-OHC on intercellular and intracellular cholesterol homeostasis in astrocytes and neurons.

## Materials and Methods

### Reagents and Materials

27-Hydroxycholesterol was provided by American Santa Cruz, and 27-OHC (10 mg) was completely dissolved in a suitable amount of ethanol, then divided equally in centrifuge tubes, with nitrogen gas blowing dry, and finally preserved at −80°C. The 27-OHC working solution contains 0.16% ethanol (v/v). Filipin III was from Cayman Chemical (#70440, Ann Arbor, MI, United States). Primary antibodies for Western blot included mouse anti-ABCA1 (Abcam, ab66217), rabbit anti-ABCG1 (Abcam, ab52617), mouse anti-Caveolin-1 (Abcam, ab17052), rabbit anti-Apolipoprotein E (Abcam, ab52607), mouse anti-LDLR (Millipore, MABS26), rabbit anti-ACAT1 (Abcam, ab168342), rabbit anti-CYP46A1 (Sigma–Aldrich, SAB2100523), mouse anti-KDEL (Santa Cruz, sc-58774), rabbit anti-flotillin (Abcam, ab41927), rabbit anti-SR-B1 (Abcam, 52629), mouse anti- N Cadherin (Abcam, ab98952), mouse anti-Aβ (Abcam, ab126649), and rabbit anti-LRP1 (Abcam, ab92544). Goat anti-mouse (#14709) and rabbit (#7074) biotinylated secondary antibodies were from Cell Signaling Technology. Antibodies for immunofluorescence included anti-CYP46A1 (Abcam, ab82814), goat anti-mouse (Abcam, ab150120), and goat anti-rabbit (Abcam, ab96899).

### Cell Culture

SH-SY5Y human neuroblastoma cells were obtained from Peking Union Medical College Cell Resource Center and C6 rat glial cells were obtained from Cell Bank, Shanghai Institutes for Biological Sciences. Cells were grown in Dulbecco’s modified Eagle’s medium (DMEM) supplemented with 10% fetal bovine serum (FBS) and penicillin (100 U/ml)/streptomycin (100 U/ml) at 37°C in an atmosphere of 5% CO_2_. The co-cultured method of neuronal SH-SY5Y and astrocytic C6 cells using a trans-well system was the same as before and cells were treated with 27-OHC (0, 5, 10, and 20 μM) for 24 h. The equivalent volume of DMEM with 0.16% ethanol (v/v) was used as the control group. The 27-OHC intervention concentrations had been screened in our previous studies (An et al., 2017; Chen et al., 2019).

### Lipid Extraction

After 27-OHC treatment for 24 h, the co-cultured SH-SY5Y cells and C6 cells were harvested and resuspended in PBS, respectively. Then, the cells were divided into two aliquots. One aliquot was used for protein determination by bicinchoninic acid (BCA) protein assay kit (Pierce Biotechnology, United States). The other aliquot was subjected to lipid extraction. Briefly, 1 ml of chloroform/methanol (2:1 v/v) was added to the cells; after shaking and mixing thoroughly, the cell-containing solution was incubated for 15 min. Then, 100 μl of ultrapure water was added, followed by centrifuging at 10,000 × *g* for 10 min. The organic phase was collected carefully and evaporated under nitrogen. The lipid extraction obtained was used for the subsequent Amplex Red cholesterol assay.

### Amplex Red Cholesterol Assay

Cholesterol levels were quantified using the Amplex Red Cholesterol Assay kit (NO. A12216, Invitrogen, United States) according to the manufacturer’s instruction. Briefly, cell extracts were diluted in 1 × Reaction Buffer. Then, 50 μl of Amplex Red reagent containing 2 U/ml HRP, 2 U/ml cholesterol oxidase, and 0.2 U/ml cholesterol esterase was added to 50 μl of samples in 96-well plates. After incubating the reactions at 37°C for 30 min, protected from light, the fluorescence was measured in a microplate reader (Tecan, Switzerland) using excitation at 530 nm and emission detection at 590 nm. Total cholesterol (TC) content was determined by measuring the cholesterol concentration following digestion with cholesterol esterase. To measure FC, cholesterol esterase was omitted from the assay. The detected cholesterol concentrations were calculated by reference to the calibration curve and normalized to protein content. Three replicates for each sample were set and three independently repeated experiments were performed.

### Flow Cytometry

SH-SY5Y cells and C6 cells co-cultured were harvested after 20 μM 27-OHC for 24 h. After rinsing with cold PBS, cells were fixed with 4% paraformaldehyde (PFA) for 10 min. Before analysis by flow cytometry, cells were resuspended in Filipin III work solution (50 μg/ml in PBS) for 1 h. Then, cholesterol content assessment was performed using a flow cytometer (Becton, Dickinson and Company, United States). More than 1 × 10^4^ cells in each sample were used to calculate the average fluorescence intensity. The experiments were repeated three times.

### ApoE ELISA

The ApoE ELISA assay was performed using a commercialized kit (NO. SEA704Ra, Cloud-Clone Corp^®^, Texas, United States) and following the procedure recommended by the manufacturer. In short, after incubated with various concentrations of 27-OHC for 24 h, cells and medium were collected, respectively. Cell lysate and medium were incubated with 100 μl of Detection Reagent A working solution for 1 h, then for another 30 min with 100 μl of Detection Reagent B, and followed by measurement using an Epoch microplate spectrophotometry (BioTek Instruments, Inc., United States) at 450 nm immediately. ApoE concentration in cell lysates was standardized using total protein concentration. All the data were repeated three times.

### High-Performance Liquid Chromatography–Mass Spectrometry (HPLC-MS)

SH-SY5Y cells and medium were harvested after treated with 20 μM 27-OHC for 24 h and obtained cell lysate by ultrasound. To the cell pellets and medium were added 24S-OHC-D5 as an internal standard (50 μl), 24S-OHC standard (50 μl), and acid buffer [200 μl, containing 50 mM ammonium acetate, 1% formic acid (pH = 3), and methyl tert-butyl ether (1 ml)]. The resulting mixture was vortexed for 10 min. All tubes were placed at −80°C for at least 1 h, and then 500 μl of the supernatant was aspirated and dried at 35°C using a nitrogen blower. The sample was derivatized (with a mixture of niacin, 4-dimethylaminopyridine and N,N’-diisopropylcarbodiimide) and reconstituted with methanol for detection. The analyses were conducted using an LC-MS/MS (Triple Quad^TM^ 6500, AB Sciex MANUFACTURING). Analyte separation was achieved using an Eclipse Plus C18 RRHD column (1.8 μm, 2.1 × 100 mm) from Agilent. Mobile phases A and B consisted of H_2_O containing 0.1% formic acid and methanol containing 0.1% methane acid, respectively. The pump gradient (0.3 ml/min) was designed as follows: a transition from 90% A to 100% B over 10 min, followed by 1 min at 100% B and equilibration at 90% A. The retention time of 24S-OHC was at 8.87 min.

### Cholesterol Depletion in Plasma Membrane

To observe cellular cholesterol content and explore the mechanism of CYP46A1 transposition, cholesterol depletion in the plasma membrane (PM) was conducted using methyl-beta-cyclodextrin (M-β-CD) (McCauliff et al., 2011; Wilhelm et al., 2019). Briefly, after discarding the medium, the cells were washed twice with cold PBS and incubated in an M-β-CD work solution (10 mM, in PBS) at 37°C, followed by washing cells twice every 2 min. Cells were fixed and stained.

### Filipin Staining

The cholesterol in C6 cells and SH-SY5Y cells were stained using the Filipin III, an established tool for cholesterol visualization. Prepare a stock solution of Filipin III (5 mg/ml) in dimethylsulfoxide (McCauliff et al., 2011). This solution should be divided into suitable portions and then preserved at −80°C and protected from light. After 27-OHC treatment of various concentrations on the 18 mm × 18 mm coverslips in 12-well plates, cells were washed in PBS twice and fixed in 4% PFA for 10 min at room temperature. Rinse the cells for 5 min using PBS, and then stain them in Filipin III work solution (50 μg/ml in PBS) for 30 min at room temperature. They were subsequently rinsed three times with PBS. Acquired images using laser confocal microscopy (Leica, Germany) with 380-nm excitation and 385- to 470-nm emission filters. The fluorescence intensity was quantified in 12 randomly selected fields per sample using Fiji software (open source). PM cholesterol quantification with Filipin III was based on region of interest (ROI) approach. Data from three independent experiments were used for statistical analysis.

### Immunofluorescence Double Staining

Cells were seeded into 12-well dish at a density of 1 × 10^6^ cells/ml and treated with 20 μM 27-OHC for 12 h. After the incubation, cells were fixed with 4% PFA for 10 min at room temperature and permeabilized with 0.25% Triton X-100 for 10 min. Cold PBS rinsing was performed between each step to remove any residual solvent. After blocking with Blocking Buffer (Solarbio, Beijing, China) for 1 h, cells were incubated with antibodies for CYP46A1 (1:1000) and KDEL (1:100) at 4°C overnight. After incubation, cells were rinsed and then incubated with Alexa Fluor 594-conjugated goat anti-mouse IgG (1:200) and DyLight 488-conjugated goat anti-rabbit IgG (1:200) for 1 h with light protection. Finally, the samples were counterstained with 10 μg/ml DAPI and preserved in antifade solution. Cells were observed under a STED confocal laser-scanning microscope (Leica, Germany). Images of co-stained cells were analyzed with the Fiji software Coloc2 plugin (open source). Co-localization was estimated by the Manders’ colocalization coefficients (MCCs), which represents the proportion of overlapping pixels in two channels.

### Subcellular Fractionation

For the CYP46A1 localization experiment, the endoplasmic network and PM fractions were extracted using the Endoplasmic network Enrichment Kit (Bestbio, Shanghai, China) and Minute TM Plasma Membrane Protein Isolation and Cell Fractionation Kit (Invent Biotechnologies, Inc., Eden Prairie, MN, United States). For endoplasmic network enrichment, 1 × 10^7^ cells were harvested, rinsed twice with ice-cold PBS, then centrifuged at 500 × *g* for 3 min to remove supernatant, and resuspended in 500 μl of Reagent A for 10 min on ice. After homogenization and centrifugation, the supernatant was moved to another tube, followed by centrifugation at 11,000 × *g* for 10 min. The supernatant was transferred into a new tube and was centrifuged again at 40,000 × *g* for 45 min. Add 400 μl of Reagent B to the sediment and subject it to high-speed centrifugation (40,000 × *g* for 45 min) after mixing. The precipitate was obtained and Reagent C containing protease inhibitor cocktail was added thereto and shocked violently at 4°C for 30 min. The mixture was endoplasmic network enrichment.

For PM extraction, 1 × 10^7^ cells were harvested by cell scraping, and the cells were rinsed once with cold PBS. Resuspend it in 200 μl of Buffer A and incubate it on ice for 5–10 min. After vortexing vigorously, transfer it to the filter cartridge and centrifuge it at 16,000 × *g* for 30 s. Resuspend the pellet by vigorous vortex for 10 s and centrifuge it at 700 × *g* for 1 min. Transfer the supernatant to a fresh 1.5-ml microcentrifuge tube and centrifuge it for 30 min at 16,000 × *g*. Resuspend the pellet in 200 μl of Buffer B and centrifuge it at 7800 × *g* for 5 min. Carefully transfer the supernatant to 1.6 ml of cold PBS and centrifuge it at 16,000 × *g* for 15–30 min. The pellet and PM proteins can be obtained. All centrifugation steps were performed at 4°C.

### Western Blot Analysis

After 27-OHC treatment, the cells were collected and homogenized in RIPA lysis buffer containing protease inhibitors (RIPA:PMSF = 100:1) and then centrifuged at 14,000 × *g* for 15 min at 4°C. The protein concentrations of the supernatants were then detected using a BCA protein assay kit (Pierce Biotechnology, United States) according to the manufacturer’s instructions. Protein samples (20 μg) were loaded on 10% SDS-acrylamide gels for separation by electrophoresis and then transferred to PVDF membranes. The transferred membranes were blocked using fresh 5% non-fat dry milk dissolved in Tris-buffered saline Tween-20 (TBST) at room temperature for 1 h, and then incubated with different primary antibodies including anti-ABCA1 (1:1000), anti-ABCG1 (1:5000), anti-Caveolin-1 (1:500), anti-Apolipoprotein E (1:2000), anti-ABCG4 (1:5000), anti-LDLR (1:1000), anti-ACAT1 (1:2000), anti-CYP46A1 (1:1000), anti-KDEL (1:200), anti-flotillin (1:1000), anti-SR-B1 (1:1000), anti-N Cadherin (1:1000), anti-Aβ (1:2000), and anti-LRP1 (1:20,000) overnight at 4°C. After incubation, membranes were rinsed three times for 15 min each with TBST and incubated for 1 h at room temperature in TBST containing secondary antibodies of goat anti-mouse IgG (1:2000) and goat anti-rabbit IgG (1:2000). The membrane scanning and analysis of the gray value representing protein expression were conducted by the Fusion FX imaging system (Vilber Lourmat, Marne-la-Vallée, France). β-Actin was used as a housekeeping protein, and the density of all the bands was normalized based on the control group. Each experiment was repeated over three times.

### Statistical Analysis

Data analysis was performed with Statistical Package for Social Science (SPSS) 20.0 (SPSS Inc., Chicago, IL, United States). To test for normality, we used the Kolmogorov–Smirnov test. Normally distributed data are expressed as mean and standard deviation (mean ± SD). Differences among groups were compared using one-way analysis of variance (ANOVA) and *post hoc* comparisons were evaluated using the Dunnett’s *t* tests. For non-normally distributed data sets, the Mann–Whitney *U* test was used. All the statistical tests were two-sided and a significant level was set at *P* < 0.05.

## Results

### 27-OHC Elevates Aβ Levels in SH-SY5Y Cells

**FIGURE 1:**
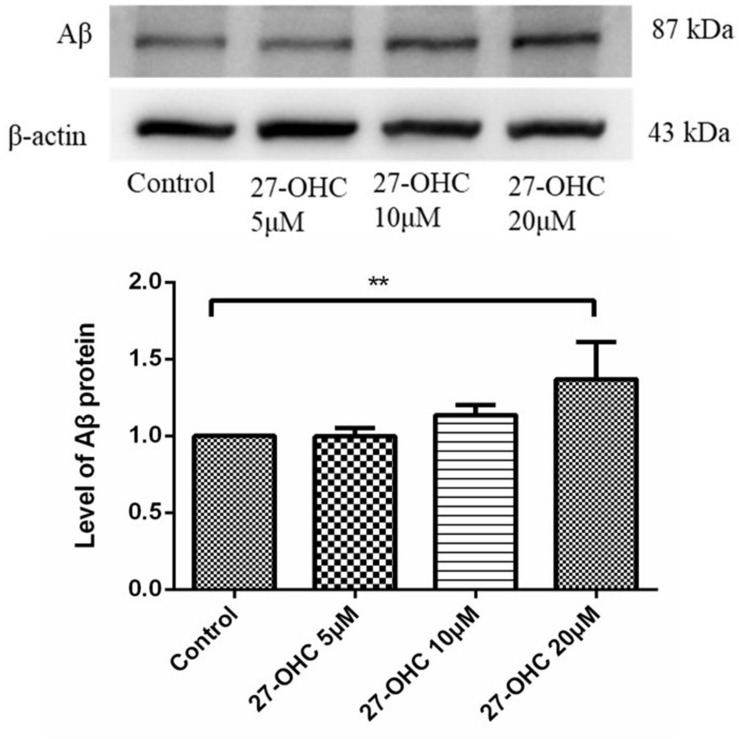
Western blot analysis of Aβ in SH-SY5Y cells with 0, 5, 10, and 20 μM 27-OHC for 24 h. Densitometric analysis was normalized with the β-actin blots. **P < 0.01 compared with the control group (one-way ANOVA).

To determine the possible pathological function of 27-OHC in AD, we detected the Aβ levels in SH-SY5Y cells after 27-OHC treatment for 24 h. Compared with the control group, treatment with 20 μM 27-OHC increased the protein level of Aβ significantly (*F* = 5.587, *P* = 0.023, Figure 1). Next, we mainly focused on the effect of 27-OHC on cholesterol metabolism.

### The Effect of 27-OHC on Cholesterol Content in SH-SY5Y Cells and C6 Cells

**FIGURE 2:**
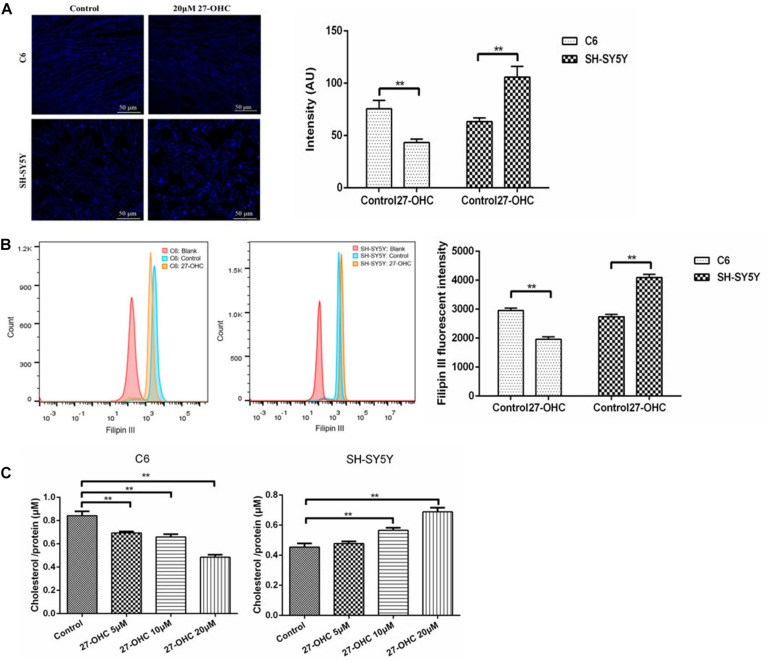
Cholesterol levels in co-cultured SH-SY5Y cells and C6 cells of the control group and 20 μM 27-OHC treatment for 24 h. **(A)** Filipin III staining under confocal microscope (scale bar: 50 μm). **(B)** Flow cytometry. **(C)** Amplex Red cholesterol assay. **P < 0.01 compared with the control group.

To investigate the effect of 27-OHC on cholesterol distribution in co-cultured SH-SY5Y cells and C6 cells, Amplex Red assay, Filipin III staining, and flow cytometry were performed. Treatment of the C6 cells with 20 μM 27-OHC resulted in a decrease of the Filipin III staining (*F* = 279.126, *P* < 0.001), while treatment of SH-SY5Y cells with 20 μM 27-OHC resulted in cholesterol accumulation (*F* = 232.018,*P* < 0.001) (Figure 2A). This was confirmed by the results of the flow cytometry (*F*_*C*__6_ = 66.996, *P*_*C*__6_ = 0.001; *F*_*SH–SY*__5__*Y*_ = 100.997, *P*_*SH–SY*__5__*Y*_ = 0.001) (Figure 2B), as well as by the Amplex Red assay (*F*_*C*__6_ = 34.515, *P*_*C*__6_ < 0.001; *F*_*SH–SY*__5__*Y*_ = 24.537,*P*_*SH–SY*__5__*Y*_ < 0.001) (Figure 2C).

### 27-OHC Facilitates Cholesterol Transport in C6 Cells and SH-SY5Y Cells

**FIGURE 3:**
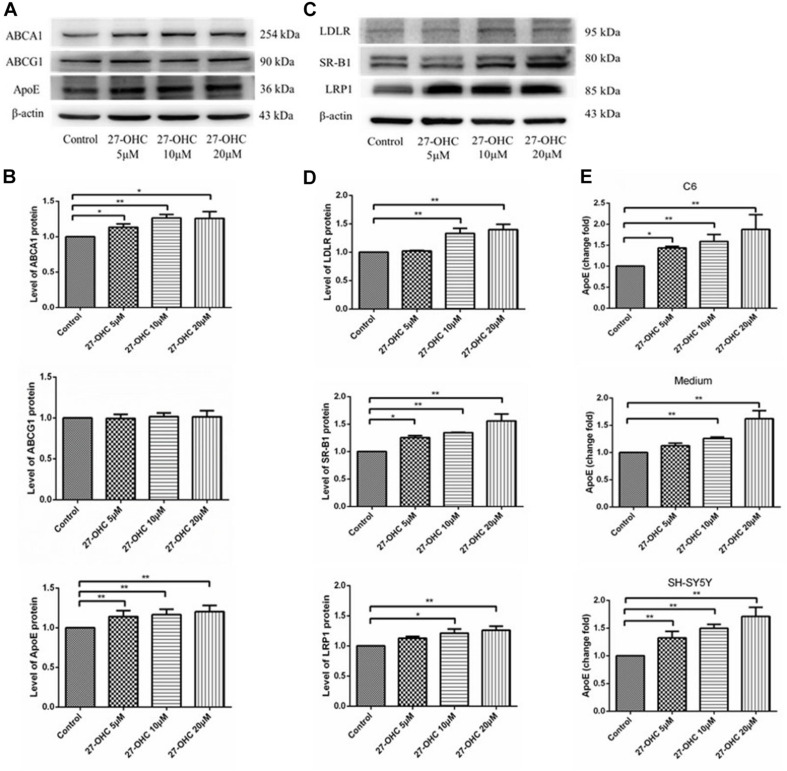
Cholesterol transport in C6 cells and SH-SY5Y cells. **(A,B)** Western blot analysis of ABCA1, ABCG1, and ApoE in C6 cells with 0, 5, 10, and 20 μM 27-OHC for 24 h. **(C,D)** Western blot analysis of LDLR, SR-B1, and LRP1 in SH-SY5Y cells after 0, 5, 10, and 20 μM 27-OHC treatment for 24 h. **(E)** ApoE content in C6 cell pellets, medium, and SH-SY5Y cell pellets using ELISA. Densitometric analysis was normalized with the β-actin blots. *P < 0.05 compared with the control group; **P < 0.01 compared with the control group (one-way ANOVA).

To assess cholesterol transport, expression levels of cholesterol transporters in C6 cells and endocytic receptors in SH-SY5Y cells were detected by Western blot. In C6 cells, ABCA1 (*F* = 18.338, *P* < 0.001) and ApoE (*F* = 7.735, *P* = 0.004) protein levels were increased after 27-OHC treatment in a dose-dependent manner. Meanwhile, no significant differences in protein expression of ABCG1 among groups were observed (*F* = 0.139, *P* = 0.934) (Figures 3A,B).

In SH-SY5Y cells, the transporters responsible for cholesterol internalization were detected using Western blot (Figures 3C,D). Results showed that 27-OHC remarkably up-regulated protein levels of LDLR (*F* = 9.726, *P* = 0.002), SR-B1 (*F* = 12.223, *P* = 0.001), and LRP1 (*F* = 5.218, *P* = 0.027).

Consistent with the findings above, ApoE levels were significantly elevated in C6 cells (*F* = 10.555, *P* = 0.004), medium (*F* = 36.351, *P* < 0.001), and SH-SY5Y cells (*F* = 23.408, *P* < 0.001), respectively (Figure 3E). The results suggested that 27-OHC facilitated cholesterol transport from astrocytes to neurons.

### 27-OHC Promotes Cholesterol Accumulation on SH-SY5Y Cell Membrane

**FIGURE 4:**
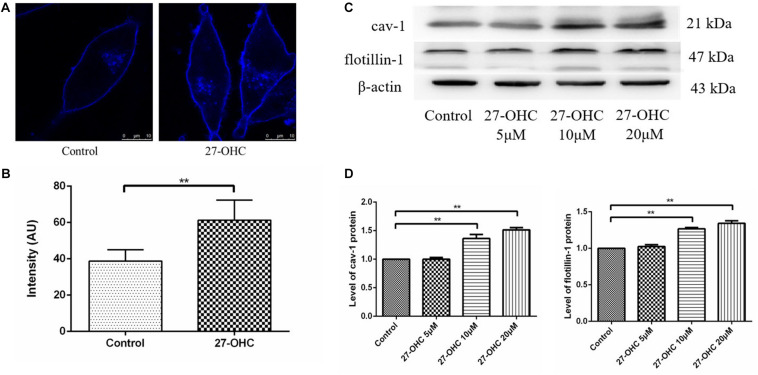
Cell membrane cholesterol assessment in SH-SY5Y cells. **(A,B)** Cholesterol observation and analysis of the control and 20 μM 27-OHC group by Filipin III staining under confocal microscope (scale bar: 10 μm). **(C,D)** Western blot analysis of cav-1 and flotillin-1 in SH-SY5Y cells after 0, 5, 10, and 20 μM 27-OHC treatment for 24 h. **P < 0.01 compared with the control group (one-way ANOVA).

Filipin III staining of SH-SY5Y cells showed higher cholesterol concentration in the 20 μM 27-OHC treatment group than the control group (Figures 4A,B).

Then, we detected cav-1 and flotillin-1 as representative protein markers that represented lipid raft in SH-SY5Y cells incubated with various concentrations of 27-OHC (0, 5, 10, and 20 μM) for 24 h (Figures 4C,D). We found that the protein levels of cav-1 (*P* < 0.001) and flotillin-1 (*P* < 0.001) were significantly up-regulated in the presence of 27-OHC in a dose-dependent manner, confirming cholesterol accumulation on PM.

### The Effects of 27-OHC on Cholesterol Esterification and Elimination in SH-SY5Y Cells

**FIGURE 5:**
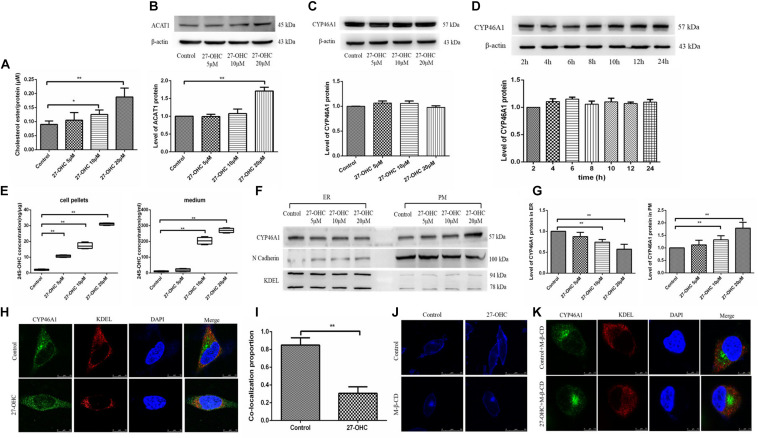
Assessment of cholesterol storage and clearance in SH-SY5Y cells. **(A)** Cholesterol ester levels in SH-SY5Y cells after 0, 5, 10, and 20 μM 27-OHC treatment for 24 h. **(B)** Western blot analysis of ACAT1 in SH-SY5Y cells after 0, 5, 10, and 20 μM 27-OHC treatment for 24 h. **(C)** Western blot analysis of total CYP46A1 in SH-SY5Y cells after 0, 5, 10, and 20 μM 27-OHC treatment for 24 h. **(D)** Western blot analysis of total CYP46A1 in SH-SY5Y cells after 20 μM 27-OHC treatment for 2, 4, 6, 8, 10, 12, and 24 h, respectively. **(E)** 24S-OHC content in SH-SY5Y cell pellets and medium after various concentrations of 27-OHC treatment for 24 h. **(F,G)** The protein level of CYP46A1 in endoplasmic reticulum (ER) and plasma membrane (PM) in SH-SY5Y cells after 0, 5, 10, and 20 μM 27-OHC treatment for 24 h. N cadherin and KDEL were used as PM and ER markers, respectively. **(H)** Immunofluorescence staining for CYP46A1 (green) and KDEL (red) confirmed that 27-OHC induced CYP46A1 translocation in SH-SY5Y cells. DAPI (blue) represented the nucleus (scale bar: 10 μm). **(I)** Colocalization analysis of CYP46A1 and KDEL. **(J)** Membrane cholesterol observation of SH-SY5Y cells in different groups. Compared with the control group, membrane cholesterol accumulation was elevated in the 20 μM 27-OHC group. 10 mM M-β-CD significantly reduced the membrane cholesterol in SH-SY5Y cells with or without 27-OHC intervention. Filipin III staining to visualize cholesterol under a confocal microscope (scale bar: 25 μm). **(K)** Immunofluorescence staining for CYP46A1 (green) and KDEL (red) confirmed that 27-OHC induced CYP46A1 translocation in SH-SY5Y cells. DAPI (blue) represented the nucleus (scale bar: 7.5 μm). *P < 0.05 compared with the control group; **P < 0.01 compared with the control group (one-way ANOVA).

To assess changes in cholesterol turnover in neurons after 27-OHC treatment, we measured the levels of cholesterol esterification and conversion to 24S-OHC. We found that treatment with 27-OHC increased the protein levels of ACAT1 (*F* = 15.298, *P* < 0.001) and the levels of CEs (*F* = 20.394, *P* < 0.001) in SH-SY5Y cells (Figures 5A,B).

Then, we evaluated the effect of 27-OHC on the hydroxylation of cholesterol to 24S-OHC. Although 27-OHC treatment at different doses (*F* = 1.482, *P* = 0.269) or times (*F* = 0.946, *P* = 0.498) did not alter CYP46A1 protein expression, there was a marked rise in 24S-OHC levels in both cell pellets and medium (*F* = 510.528, *P* < 0.001; *F* = 368.530, *P* < 0.001) (Figures 5C–E).

Since CYP46A1 mobilization from the ER to the PM could increase 24S-OHC levels, we speculated that CYP46A1 mobilization was involved in 27-OHC-induced 24S-OHC generation in SH-SY5Y cells. By cell fraction separation combined with Western blot, N cadherin and KDEL were selected as marker proteins of PM and ER, respectively (Marecic et al., 2017; Ode et al., 2017), we found that with the increase of 27-OHC treatment concentration, the protein expression of CYP46A1 gradually decreased in the ER and increased in the PM (Figures 5F,G). Consistent with this, immunofluorescence staining suggested that 27-OHC down-regulated CYP46A1 expression in the ER but up-regulated the expression in the PM (Figures 5H,I). These results indicated that 27-OHC promoted the mobilization of CYP46A1 protein from the ER to the PM, facilitating 24S-OHC release.

The above experimental results showed that 27-OHC could promote cholesterol accumulation in the PM. The distribution of CYP46A1 is also affected by the cholesterol content in PM. We used M-β-CD for cholesterol deprivation in PM to further study the mechanism of 27-OHC on CYP46A1 translocation. As shown in Figure 5J, cholesterol levels in SH-SY5Y cell membrane were effectively reduced after 10 mM M-β-CD incubation in both control group and 20 μM 27-OHC group. Then, fluorescent staining of CYP46A1 under conditions of PM cholesterol removal showed no significant changes in its intracellular localization (Figure 5K).

Based on the above experimental results, we speculated that 27-OHC could promote the translocation of cellular CYP46A1 by increasing cholesterol accumulation on the PM, instead of regulating CYP46A1 expression directly to facilitate the conversion of cholesterol to 24S-OHC.

## Discussion

There is increasing evidence that abnormalities in neuronal cholesterol homeostasis contribute to the progression of several neurodegenerative diseases (Vance, 2012; Djelti et al., 2015). Cholesterol is a pivotal component of the neuronal membrane. It influences brain function in many different ways, including membrane fluidity, vesicle trafficking, signal transduction, and formation and maintenance of synapses involved in neural plasticity (van Deijk et al., 2017). However, the deleterious effects of the overload of neuronal cholesterol have been recognized. Several exquisite experiments *in vitro* and *in vivo* have suggested that the neuronal cholesterol concentration can be a sensitive regulator of the production and amount of the Aβ peptides (Ehehalt et al., 2003). This can be explained by the findings that the β- and γ-secretase-producing Aβ peptides from its precursor are mainly localized to the lipid raft, a cholesterol-rich PM microdomain (Barrett et al., 2012). This indicates that excessive cholesterol in the brain is detrimental to nerve cell function. On the other hand, the cholesterol released from death-ruptured nerve cells also becomes a non-negligible brain cholesterol pool source.

Multiple studies have shown that hypercholesterolemia is a risk factor for AD. One possible mechanism underlying the proposed negative effect on cognitive by elevated plasma cholesterol might be attributable to the neurotoxic ability of its oxidation products. Given the correlation between 27-OHC and circulation cholesterol, 27-OHC may mediate the effect of hypercholesterolemia on the brain (Shafaati et al., 2011). In an experimental study of AD patients, Popp et al. (2012) found that the high plasma and CSF concentrations of cholesterol, 24S-OHC, and 27-OHC are associated with increased CSF concentration of soluble amyloid precursor protein forms. Moreover, in animal model experiments, Brooks et al. (2017) have demonstrated that increased circulation cholesterol in rabbits induced by high-cholesterol diet is related to a higher amount of 27-OHC accompanied by hippocampal endoplasmic reticulum (ER) impairment and mitochondrial dysfunction, whereas the effect of 27-OHC on cholesterol homeostasis in the brain is still under-researched. Considering that cholesterol exchange between astrocytes and neurons is relatively the most active, astrocytes releasing ApoE-containing lipoproteins provide neurons with cholesterol. We used co-cultured C6 cells and SH-SY5Y cells *in vitro* to assess 27-OHC function on cholesterol metabolism in nerve cells.

Experiments with the Abcg1^–/–^ and Abcg4^–/–^ mice and gene interference strategy *in vitro* (ABCG1 and ABCG4 siRNA transfections) have shown that the cholesterol efflux mechanism in astrocyte is mainly the responsibility of ABCA1 and ABCG1 (Pfrieger and Ungerer, 2011; Chen et al., 2013) and neurons take up cholesterol-containing lipoproteins via receptors such as LDLR, LRP1, and SR-B1 (Brown and Goldstein, 1986; Panchoo and Lacko, 2018). In this study, 27-OHC up-regulated the protein expression of ABCA1, ApoE in astrocytes, and LDLR, LRP1, and SR-B1 in neurons, consistent with the result in hippocampal neurons (Rapp et al., 2006). The cascade of 27-OHC on cholesterol efflux from astrocytes and influx into neurons ultimately contributes to neuronal cholesterol overload. Fluorescent staining observed with a confocal microscope and the expression of two key lipid raft proteins (flotillin-1 and cav-1) in this work demonstrated that the increased amount of cholesterol was concentrated on PM. This might explain, from one aspect, the neurotoxic effect of elevated 27-OHC levels on AD-related pathological changes such as Aβ deposition, hyperphosphorylated tau tangles, and cell apoptosis in the brain (Huang et al., 2016; Panahi et al., 2016; Chang et al., 2017). Meanwhile, cholesterol esterifying activity was enhanced in neurons after 27-OHC treatment through the up-regulated protein expression of ACAT1, also called SOAT1 (Zhu et al., 2015), a rate-limiting enzyme responsible for the esterification process of cholesterol mainly expressed in neurons.

We also demonstrated that intervention with various concentrations of 27-OHC increased the production of 24S-OHC significantly in both cells and medium, which is a specific way for neurons to handle surplus cholesterol. While no significant change in the expression of CYP46A1 was detected, the mobilization of CYP46A1 from ER to PM was stimulated by the 27-OHC-induced increase in membrane cholesterol, resulting in an increase in 24S-OHC (Sodero et al., 2012).

Based on the results of subsequent experiments after the removal of cholesterol from the PM, we speculated that intracellular translocation of CYP46A1 promoting cholesterol elimination as 24S-OHC was a feedback mechanism of neurons to restore cholesterol homeostasis. In both Cyp46a1 knockout mice and the Cyp46a1 transgenic mice, cholesterol content was found to remain unchanged due to the corresponding regulation of cholesterol synthesis (Shafaati et al., 2011; Maioli et al., 2013). Bryleva et al. used an ACAT1 gene knockout mouse model finding that high cholesterol levels in the brain leads to decreased cholesterol synthesis and more production of 24S-OHC (Shafaati et al., 2011; Maioli et al., 2013). Taking these together, our results indicated that the increased flow of 27-OHC into the brain interfered with cholesterol transport between brain cells, thereby contributing to cholesterol accumulation in neurons, providing evidence that 27-OHC plays a deleterious role in cholesterol homeostasis in the brain, which is one of the AD-like pathologies.

It is undeniable that there exist several limitations in the present research. First, this research focused on the cholesterol metabolism of astrocytes and neurons without considering the possible effects of other cells, such as microglia (El-Sayyad, 2015). Second, the regulation of intercellular and intracellular cholesterol transport is a continuous process, so changes in the expression of the detected indicators may not be enough to reflect the dynamics of cholesterol homeostasis. Further, there are potential metabolic differences between cell lines and *in vivo* cells. The interpretation of the present study results should be made with caution when extending the significance to real cells. Nonetheless, our results provided evidence for the correlation between peripheral and brain cholesterol metabolism, with 27-OHC acting as a link.

## Conclusion

We found that elevated 27-OHC has a marked impact on cholesterol metabolism in neurons, which is associated with the hyperactivity of cholesterol traffic between nerve cells. In addition, enhanced conversion to 24S-OHC catalyzed by CYP46A1 approaching the PM is feedback regulation for excessive cholesterol increase in neurons. These findings suggest that excessive 27-OHC plays a role in the interaction of intracerebral and peripheral cholesterol metabolism pools, inducing cholesterol accumulation in neurons, causing nerve cell dysfunction and aggravating cognitive decline.

## Data Availability Statement

The original contributions presented in the study are included in the article/supplementary material. Further inquiries can be directed to the corresponding author.

## Author Contributions

RX conceptualized and designed the study and obtained funding. YSW conducted the experiments and drafted the manuscript. XNZ, TW, WL, LJW, LH, and MWJ participated in the interpretation of the results, review, and editing. All authors agreed to be accountable for the content of the work and approved the final manuscript.

## Conflict of Interest

The authors declare that the research was conducted in the absence of any commercial or financial relationships that could be construed as a potential conflict of interest.
